# Is there a role of hepatic enzymes in the management of penetrating hepatic injuries?

**DOI:** 10.1186/1757-7241-21-S1-S19

**Published:** 2013-05-28

**Authors:** JR Pallett, A Gibbon, JW Keep

**Affiliations:** 1 Kings College Hospital, UK

## Background

The association of hepatic enzymes and blunt hepatic trauma has been widely researched and a general consensus exists on the role of non-operative management of these injuries.[1] Penetrating liver injuries are less common and subsequently, management of the hemodynamically stable patient with a penetrating liver injury remains a greater challenge.

## Methods

A retrospective cohort study of all penetrating and blunt liver injuries at a Level 1 Major Trauma Centre (King’s College Hospital NHS Foundation Trust) was undertaken. Cases were defined by having radiological evidence of liver trauma following injury. Initial aspartate transanimase (AST) levels were documented (normal 10-50 IU/L) along with intervention (non-operative or operative) and outcome.

## Results

35 cases of penetrating trauma (3 gunshot wounds, 32 stabbings) over a 48 month period were identified. ISS mean 14.77 (4-36), Length of Stay (LOS) mean 13.97 days (3-54), age mean 26.25yrs (17-52), 97.14% male. 28.57% (n=10) had abnormally elevated AST levels (51 to 188IU/L) at time of presentation to the ED. Of those with elevated initial AST levels, 70% (n=7/10) had an emergency laparotomy compared with 28% (n=7/25) of cases of penetrating trauma with a normal AST level. Initial raised AST was therefore more likely associated with need for operative management (p=0.022). Of interest, of the three cases with raised AST without laparotomy, one developed a bile leak requiring CBD stent and another developed a pseudoaneurysm and self-discharged prior to further evaluation. In contrast, 28 cases of blunt trauma over a 29 month period were identified with an ISS mean of 26.32 (4-66), LOS mean 24.27 (1-114), mean age of 33.34yrs (1-58.1). 89% (n=25) of these blunt liver injuries had elevated AST levels (69 to 3913IU/L) and only 2 cases proceeded to laparotomy which was for associated injuries and not liver trauma.

## Conclusion

There study suggests that raised (AST) is associated with having a laparotomy rather than conservative management in penetrating hepatic injuries and justifies further research into the diagnostic value of hepatic enzymes in the stable patient with penetrating injury.
